# Prevalence of *Helicobacter pylori* resistance to certain antibiotics at An-Najah University Hospital: a cross-sectional study

**DOI:** 10.1038/s41598-024-63982-0

**Published:** 2024-06-24

**Authors:** Qusay Abdoh, Mohammad Alnees, Lubna Kharraz, Khubaib Ayoub, Abdalaziz Darwish, Mahdi Awwad, Duha Najajra, Jana Khraim, Wafaa Awad, Aesha Sbaih, Safaa Turman, Nizar Abu Hamdeh

**Affiliations:** 1 Faculty of Medicine, An-Najah National University, Nablus, Palestine; 2 Division of Gastroenterology, An-Najah National University Hospital, Nablus, Palestine; 3 Department of Internal Medicine, Specialized Araby Hospital, Nablus, Palestine; 4 Global Clinical Scholars Research Training Program, Harvard Medical School Postgraduate Medical Education, Boston, USA; 5 Department of Medicine, Faculty of Medicine and Health Sciences, An-Najah National University, Nablus, Palestine

**Keywords:** Antibiotic resistance, Antimicrobial susceptibility testing, *Helicobacter pylori*, Ciprofloxacin, Microbiology, Structural biology, Gastroenterology, Medical research

## Abstract

Antibiotic resistance among bacteria is recognized as the primary factor contributing to the failure of treatment. In this research, our objective was to examine the prevalence of antibiotic resistance in *H. pylori* bacteria in Palestine. We enlisted 91 individuals suffering from dyspepsia, comprising 49 females and 42 males. These participants underwent esophagogastroduodenoscopy procedures with gastric biopsies. These biopsies were subsequently subjected to microbiological assessments and tested for their susceptibility to various antimicrobial drugs. Among the 91 patients, 38 (41.7%) exhibited the presence of *H. pylori*. Notably, Ciprofloxacin displayed the highest efficacy against *H. pylori*, followed by Levofloxacin, Moxifloxacin, and Amoxicillin, with resistance rates of 0%, 0%, 2.6%, and 18.4%, respectively. On the contrary, Metronidazole and Clarithromycin demonstrated the lowest effectiveness, with resistance percentages of 100% and 47.4%, respectively. The outcomes of this investigation emphasize that *H. pylori* strains within the Palestinian patient group exhibit substantial resistance to conventional first-line antibiotics like clarithromycin and metronidazole. However, alternative agents such as fluoroquinolones and amoxicillin remain efficacious choices. Consequently, we recommend favoring quinolone-based treatment regimens for *H. pylori* infections and adopting a more judicious approach to antibiotic usage among the Palestinian population.

## Introduction

*Helicobacter pylori (H. pylori)* is a type of *microaerophilic*, gram-negative bacterium that is present throughout the world, particularly in developing nations. It functions as a prevalent pathogenic *microorganism* and has been identified over the past thirty years as a notable contributor to gastrointestinal ailments, which encompass *peptic* ulcers, persistent *gastritis*, *gastric* cancer, and *gastric mucosa-associated lymphoid tissue (MALT) lymphoma*. The classification assigned by the World Health Organization is that of a class I carcinogen. Furthermore, *H. pylori* infection has been linked to irregularities within the nervous, circulatory, and *hematopoietic* systems. The eradication of *H. pylori* infection assumes a pivotal role in the prognosis of these conditions. Clinical investigations underscore that removing the infection yields tangible advantages by fostering the healing of *gastric* mucosa and reducing the factors predisposing to MALT lymphoma and *gastric* cancer. Thus,* H. pylori* eradication holds significant importance in clinical practice^1–6^.

The presently endorsed primary approach for eliminating *H. pylori* involves a triple therapy that centers around clarithromycin, coupled with a proton pump inhibitor (PPI) and either amoxicillin or metronidazole. This treatment protocol yields an eradication success rate ranging from approximately 70 to 85%. Nonetheless, instances of treatment ineffectiveness are primarily attributed to poor adherence to the regimen and the escalating incidence of antibiotic resistance, notably the rising levels of clarithromycin resistance on a global scale^7^. The utilization of multiple antibiotics in *H. pylori* treatment has spurred the emergence of bacterial antibiotic resistance, albeit with distinct patterns varying across regions^8^. The infection rates with *H. pylori* tend to be higher in developing countries compared to developed ones. Consequently, understanding resistance levels within individual countries and populations is crucial. The objective of this study is to discern the antibiotic resistance pattern of *H. pylori* in previously untreated patients in Palestine.

## Methods

### Study population

The study targeted all adult patients who underwent upper GI endoscopy for various me*dical reasons at NNUH between July 2016 and January 2017 and agreed to participate by signing the consent form.*

- *Inclusion criteria*:
  All participants were aged 18 years or older and had undergone upper endoscopy at An-Najah National University Hospital during the study period.
- *Exclusion criteria*:
  Patients were excluded if they had made previous attempts to eradicate *H. pylori*, used antibiotics or proton pump inhibitors within the last 2 weeks before endoscopy, or had undergone previous gastric surgery.

### Sampling

Ninety-one patients with diverse gastrointestinal symptoms representing various age groups and genders were included. Samples were collected from the Endoscopy Department of Al-Najah National University Hospital in Nablus. Prior informed written consent was obtained from each patient.

#### Sample size calculation

The sample size was calculated using Cochrane formula for sample size calculation in prevalence studies.

$${\text{n}}_{{\text{o}}} = \frac{{\left( {{\text{Z}}_{{\upalpha /2}}^{2} } \right){\text{p}}\left( {1 - {\text{p}}} \right)}}{{\Delta^{2} }}$$

Considering 95% confidence level (z = 1.96), estimation error (E = 0.10), and the prevalence (*p* = 0.5), the calculates sample size is n = 86 samples. Considering drop out 5% the required sample size is 90 samples.

### Specimen collection

During endoscopic diagnosis, Dr. Qusay Abdoh, a gastroenterologist at An Najah National University Hospital, collected *antral* and *corpus* mucosal biopsies from the stomach. The *antral* biopsy was taken from the lower part adjacent to the *pylorus*, while the *corpus* biopsy was obtained from the stomach’s body region responsible for producing *gastric* juices. Biopsies were transported in 2 ml Brain–heart infusion broth with ice and processed within four hours of collection to ensure sample integrity.

### Biopsy culturing

Biopsy samples were minced and homogenized in sterile petri dishes near a bunsen burner. The minced biopsy, along with 0.2 ml of transport media, was incubated on Brain–heart infusion agar media plates supplemented with 5% sheep blood and *Helicobacter pylori* selective supplement (Dent) for primary isolation. Cultures were incubated at 37 °C under *microaerophilic* conditions. Plates were examined for positive growth at 7 days intervals. Positive growth was characterized by tiny, glistering, translucent or gray colonies with intact edges.

### Biochemical tests of *H. pylori*

- *Urease test*:
  Grown colonies were inoculated on urea agar slants containing phenol red. The color change from yellow to pink was observed before and after 1 h and 24 h of incubation.
- *Catalase test*:
  Hydrogen peroxide (3%) was added to grown isolated colonies on a sterile slide. The production of gas bubbles within 20–30 s indicated a positive reaction.
- *Oxidase test*:
  An isolated colony was transferred into an oxidase strip. A positive reaction was indicated by an intense deep purple color appearing within 5–10 s.
- *Antibiotic susceptibility test:*
  Positive *H. pylori* isolates were inoculated in brain–heart infusion broth supplemented with 5% sheep or human serum and incubated at 37 °C for 24 h. Inoculated broth was spread on Muller-Hinton agar plates supplemented with 5% sheep blood. Antibiotic disks were placed on the plates and incubated under *microaerophilic* conditions for 7 days. The diameters of inhibition zones were measured using a ruler(mm).

### Statistical analysis

All statistical analyses were completed using SPSS version 27 (IBM Corp., Armonk, NY, USA) and R version 3.5.1 (R Foundation for Statistical Computing, Vienna, Austria). A *p* value lower than 0.05 was considered the threshold for statistical significance.

### Ethical approval

Ethical approval was taken from the *institutional review board (IRB) of An-Najah National University*.

### Informed consent

Informed consent was obtained from all individual participants included in the study. and conducted according to the Helsinki Declaration of Human Rights.

## Results

A total of 91 individuals experiencing *dyspepsia* were enrolled, consisting of 49 females and 42 males. Their ages spanned from 19 to 82 years, with an average age of 55. These participants underwent diagnostic upper gastrointestinal endoscopy at the endoscopy department of Al-Najah National University Hospital in Nablus, Palestine. Multiple *gastric* biopsy samples were extracted from both the *antrum* and the body of the stomach. The identification of *H. pylori* within the *gastric* biopsy specimens was carried out using tests for urease production and cultural examination.

### Detection of *H. pylori* occurrence among dyspeptic patients

The occurrence of *H. pylori* was observed in 38 out of the 91 patients (41.7%), with 23 cases from males and 15 cases from females. Table 1 illustrates the distribution of infection percentages among various diagnoses. Notably, the highest infection percentage was observed in patients with *duodenal* ulcers, where all three cases (100%) were infected. Among patients with gastritis, 20 cases (54%) were infected, followed by 12 cases (41.1%) of individuals with a normal endoscopic appearance, and 3 cases (30%) of patients with *gastric* ulcers. The remaining twelve patients with different endoscopic features did not exhibit *H. pylori* infection.

**Table 1:** Occurrence of *H. pyloi* among dyspeptic patients.

Endoscopic diagnosis	No. of cases	Infection cases no	%
*Duodenal* ulcer	3	3	100
*Gastritis*	37	20	54
*Gastric* ulcer	10	3	30
Healthy	29	12	41.1
Different Dx	12	–	–
Total	91	38	41.7

### Antibiotic sensitivity of *H. pylori*

The customary disk diffusion technique was utilized to evaluate the antibiotic resistance profile of *H. pylori* strains in relation to six distinct antibiotics. Table 2 reveals that *ciprofloxacin* and *levofloxacin* were the most effective antibiotics against *H. pylori* isolates, with all isolates showing sensitivity to them. *Moxifloxacin* followed with only one isolate showing resistance. In contrast, *amoxicillin* exhibited resistance in seven isolates. Conversely, *metronidazole* emerged as the least effective antibiotic, with all thirty-eight isolates being resistant to it. *Clarithromycin* followed with eighteen isolates exhibiting resistance.

**Table 2:** Antibiotic susceptibility of *H. pylori*.

Antibiotic	No. of sensitive isolates	No. of resistant isolates
*Ciprofloxacin*	38	0
*Levofloxacin*	38	0
*Moxifloxacin*	37	1
*Amoxicillin*	31	7
*Clarithromycin*	20	18
*Metronidazole*	0	38

## Discussion

The strategy for addressing *H. pylori* infection differs markedly from the procedures employed for other contagious ailments. For example, the treatment of *H. pylori* necessitates customization, ideally guided by local and individual antibiotic resistance profiles^9^. Our understanding of the underlying mechanisms driving treatment resistance in *H. pylori* has advanced considerably. This involves a range of mechanisms, including chromosomally encoded alterations and physiological changes that affect drug uptake, efflux, as well as biofilm and coccoid production^10^. In Palestine, gaining insights into the initial rates of resistance exhibited by *H. pylori* strains against antibiotics such as amoxicillin, metronidazole, clarithromycin, ciprofloxacin, levofloxacin, and moxifloxacin holds paramount importance. This is particularly significant given the significant public health concerns associated with antibiotic resistance concerning *co*The increasing prevalence of multi-drug-resistant (MDR) *H. pylori* strains, resilient to various drug categories, presents a disconcerting challenge amid the global antibiotic resistance surge^10^. Helicobacter pylori infection affects over 50% of the global population, yet only a small fraction of those infected develop serious gastroduodenal conditions such as duodenal ulcer, gastric ulcer, and gastric adenocarcinoma. These MDR strains, constituting over 40% of infections in specific regions, confront effective treatment approaches^11^. The development of MDR in *H. pylori* arises from mechanisms including concurrent mutations conferring resistance to diverse drug families, leading to cumulative MDR profiles^10,12^. Additional mechanisms involve the activation of expulsion systems for drug removal and potential changes in membrane protein or *lipopolysaccharide* expression, affecting drug uptake. The formation of biofilms, structured bacterial communities enclosed in a matrix, amplifies resilience and resistance. Unlike free-floating bacteria, biofilm-linked *H. pylori* can endure, potentially facilitating antibiotic resistance evolution. While *H. pylori* predominantly exists as free-floating in the stomach, biofilms might contribute to resistance and alternative transmission routes^10,13–16^.

According to a recent clinical practice update study, the choice of treatment regimen for eradicating *H. pylori* infection should be influenced by the local prevalence of *clarithromycin* resistance and the patient's history of macrolide use^17^. For initial treatment, *quadruple* therapies such as *bismuth quadruple* and concomitant regimens are recommended. In regions with a low incidence of *clarithromycin* resistance and among individuals who haven’t previously used *macrolides*, a 14 days triple therapy containing *clarithromycin* is suggested. However, the effectiveness of sequential therapy against *clarithromycin*-resistant *H. pylori* strains is conflicting, leading to a general reluctance towards its use. In cases where first-line treatments fail, second-line options encompass *levofloxacin*-containing triple therapy and *bismuth quadruple* therapy. Incorporating *probiotic* supplementation aims to mitigate antibiotic-related adverse effects. Recent evidence endorses the existing guideline recommendations for *Helicobacter pylori* infection treatment, reinforcing their relevance and validity^17^.

The findings indicated that all the strains examined in this investigation displayed resistance to metronidazole^18^. This level of resistance aligns with observations from developing nations, where metronidazole resistance has been reported to range from 17 to 100%^17^. However, in developed countries, most reports indicate resistance rates of 15.8–40% for *H. pylori* strains^18^. Additionally, 18.4% of the isolates were resistant to *amoxicillin*. This increase in resistance rates to *metronidazole* and *amoxicillin* could be attributed to the increased colonization of the stomach with antibiotic-resistant plasmids transferred from other bacteria.

Clarithromycin is a macrolide antibiotic commonly employed as part of combination treatments for *H. pylori* infection. Nevertheless, the emergence of clarithromycin resistance has emerged as a primary cause of treatment ineffectiveness^19^. Resistance in 47.4% of our isolates resembles data from one European study^19^. In contrast, resistance rates were notably lower in the USA (29%), Mexico (28.2%), Japan (30%), China (38%), and Turkey (47.5%)^19^. Since *clarithromycin* is used to treat infections outside the gastric tract, its prevalence in *H. pylori* resistance is on the rise.

Resistance rates of *ciprofloxacin, levofloxacin,* and* moxifloxacin* in our isolates were 0%, 0%, and 2.6%, respectively^19^. Resistance to *levofloxacin* remains low globally, less than 19%^20^. According to prevailing global recommendations and an extensive meta-analysis, treatment approaches involving non-bismuth quadruple therapies lasting 10–14 days and vonoprazan-based triple therapies lasting 7 days are presently advised for *H. pylori* infection. These regimens achieve eradication rates of approximately 90%, even in areas where antimicrobial-resistant bacteria are prevalent^21^.

## Conclusion

In this study carried out in Palestine researchers conducted an investigation, into the occurrence and susceptibility of *H. Pylori* among patients with digestive discomfort. The results unveiled a prevalence of *H. Pylori* infection at 41.7% within the study participants with an occurrence observed among individuals diagnosed with *duodenal* ulcers and *gastritis*. Notably the resistance rates to two prescribed antibiotics, *metronidazole* and *clarithromycin* were alarmingly high at 100% and 47.4% respectively. On the hand *ciprofloxacin* and *levofloxacin* exhibited effectiveness as all tested samples showed sensitivity to these antibiotics. Moreover, *amoxicillin* resistance was detected in 18.4% of cases providing insights into the landscape of resistance, in *H. Pylori*.

Based on these findings it is recommended to be cautious when treating individuals infected with *H. Pylori*, in Palestine. Due to the rates of resistance observed it is advised to exercise caution when prescribing *metronidazole* and *clarithromycin* as part of the treatment plan. Instead considering the effectiveness of *ciprofloxacin* and *levofloxacin* these antibiotics could be suitable options for combination therapy. Adding *ciprofloxacin* or *levofloxacin* to the treatment protocol with *amoxicillin* or other appropriate antibiotics may increase the chances of successful eradication. Additionally considering the concerning increase in resistance a comprehensive approach is necessary. It is crucial to monitor patterns of antibiotic resistance regularly evaluate treatment outcomes and prioritize patient education on adhering to prescribed regimens. This study underscores the importance of tailoring treatment approaches based on resistance profiles and emphasizes the critical need for ongoing research and vigilance to effectively mitigate the impact of antibiotic resistance on both *H. pylori* management and public health.

## Limitation

In our study on Helicobacter pylori resistance at An-Najah University Hospital, we encountered challenges. Firstly, it was difficult to standardize the size of the inoculum because the bacterias growth rate was slow and it had requirements. Secondly although we carefully measured the zone of inhibition, we did not clearly specify the sizes and cutoff points which could impact how the results are interpreted. Thirdly our ability to measure Minimum Inhibitory Concentration (MIC) was hindered by the unavailability of the E test method. Moreover, despite our efforts incorporating ATCC strains for quality control purposes proved unsuccessful due, to contamination and degradation issues. These challenges underscore the importance of refining our methods and exploring approaches, in studies.

## Data Availability

The data sets supporting the results of the current research are available from the corresponding authors upon request.
